# A Polymer-Based Magnetic Resonance Tracer for Visualization of Solid Tumors by ^13^C Spectroscopic Imaging

**DOI:** 10.1371/journal.pone.0102132

**Published:** 2014-07-09

**Authors:** Yoshikazu Suzuki, Mitsuru Iida, Iwao Miura, Toshiro Inubushi, Shigehiro Morikawa

**Affiliations:** 1 Diagnostic Division, Otsuka Pharmaceutical Co., Ltd., Tokushima, Tokushima, Japan; 2 Pharmaceutical Division, Otsuka Pharmaceutical Co., Ltd., Tokushima, Tokushima, Japan; 3 Biomedical MR Science Research Center, Shiga University of Medical Science, Otsu, Shiga, Japan; University of Pécs Medical School, Hungary

## Abstract

Morphological imaging precedes lesion-specific visualization in magnetic resonance imaging (MRI) because of the superior ability of this technique to depict tissue morphology with excellent spatial and temporal resolutions. To achieve lesion-specific visualization of tumors by MRI, we investigated the availability of a novel polymer-based tracer. Although the ^13^C nucleus is a candidate for a detection nucleus because of its low background signal in the body, the low magnetic resonance sensitivity of the nucleus needs to be resolved before developing a ^13^C-based tracer. In order to overcome this problem, we enriched polyethylene glycol (PEG), a biocompatible polymer, with ^13^C atoms. ^13^C-PEG40,000 (^13^C-PEG with an average molecular weight of 40 kDa) emitted a single ^13^C signal with a high signal-to-noise ratio due to its ability to maintain signal sharpness, as was confirmed by *in vivo* investigation, and displayed a chemical shift sufficiently distinct from that of endogenous fat. ^13^C-PEG40,000 intravenously injected into mice showed long retention in circulation, leading to its effective accumulation in tumors reflecting the well-known phenomenon that macromolecules accumulate in tumors because of leaky tumor capillaries. These properties of ^13^C-PEG40,000 allowed visualization of tumors in mice by ^13^C spectroscopic imaging. These findings suggest that a technique based on ^13^C-PEG is a promising strategy for tumor detection.

## Introduction

Magnetic resonance imaging (MRI) is a powerful tool for non-invasive exploration of internal body structures with excellent spatial and temporal resolution. Its power is derived from the widely distributed, robust proton signaling from the enormous amounts of water in the body, which enables rapid and precise depiction of morphology. On the other hand, the detection of lesion sites with a specific tracer, in which the modalities of positron emission tomography (PET) and single-photon emission computed tomography (SPECT) play an important role, is also a powerful tool in imaging diagnostics, especially in the field of tumor detection. However, no tracer-based method for tumor detection using MRI is presently in wide use, possibly because only a few strategies are currently available. Therefore, the further development of tracer-based strategies with general versatility is anticipated.

The ^13^C nucleus appears to be a promising candidate for detection in tumor imaging because its background signal is much lower than that of protons. However, ^13^C spectroscopic imaging will not be possible until the inherently low sensitivity of ^13^C nuclei is resolved. The dynamic nuclear polarization (DNP) technique, which enables marked enhancement of the MR signal from ^13^C nuclei with the induction of a hyperpolarized state in the nuclei in the liquid phase [1], [2], is an excellent way to overcome the low sensitivity of ^13^C nuclei. Using this technique, non-invasive tumor detection has been achieved *in vivo* as a result of the altered pH in tumor tissue [3] or the altered pyruvate metabolism in tumor cells [4]. This technique also enables spectroscopic evaluation of the tumor response to anti-tumor therapies [5], [6], [7]. Interestingly, it has been also reported that the hyperpolarized ^13^C glutamate generated by the DNP technique can be a metabolic biomarker of the mutational status of isocitrate dehydrogenase 1 gene in glioma *in vivo* by detecting the activity of the branched-chain amino acid transaminase 1 [8]. Parahydrogen-induced polarization (PHIP), which utilizes parahydrogen to induce the hyperpolarization of ^13^C nuclei, is another hyperpolarization technique that has been developed [9] and has been demonstrated to have utility in tumor detection [10]. Although a limitation of these hyperpolarization techniques is a rapid decrease in the enhanced signal due to the short T_1_ lifetimes of ^13^C nuclei, a method to sustain the hyperpolarized ^13^C magnetization using long-lived states of enhanced proton polarization has been developed [11]. More recently, it has been demonstrated that hyperpolarization of a deuterated ^13^C-labeled glucose enables the *in vivo* metabolic imaging of glucose in tumor by ^13^C spectroscopy due to the prolonged lifetime of the hyperpolarized state of the ^13^C atom in the double-labeled glucose [12]. Using this technique, tumor response to treatment of a chemotherapeutic agent has been clearly observed. With the future application of such improvements to overcome the short lifetime, and together with the hardware improvement such as equipping a ^13^C cryoprobe [13] and also with the data processing technology enabling fast and efficient spectroscopic imaging [14], hyperpolarization methods for ^13^C spectroscopic imaging will be a promising advancement in the field of tumor detection and tumor metabolic imaging.

In this study, we examined an alternative method for tumor detection using ^13^C spectroscopic imaging. Our method utilizes a ^13^C-enriched polymer to overcome the low sensitivity of the ^13^C nucleus rather than the hyperpolarization phenomenon. It is well recognized that macromolecules selectively extravasate into tumors because of the enhanced leakiness of tumor capillaries [15], [16]. Thus, a macromolecular formulation would be a suitable candidate for a tracer aimed at tumor detection. A ^13^C tracer that could be used as a macromolecular formulation without using the hyperpolarization technique should satisfy 4 conditions. First, the agent should contain a large number of carbon atoms with identical chemical environments in order to achieve a sufficient ^13^C signal. Second, the molecules of the tracer should be sufficiently flexible in aqueous solution such that their signaling remains sufficiently strong, regardless of their mass. Third, the ^13^C chemical shifts in the tracer should be clearly distinguishable from the large and broad signals derived from endogenous fat *in vivo*. Fourth, the tracer should have high biocompatibility with human tissue to enable diagnosis in humans.

Among the candidate agents for use as a ^13^C tracer, we identified polyethylene glycol (PEG), which has a unique chemical structure consisting of a repetition of -CH_2_CH_2_O-, as a compound satisfying these conditions. On the basis of the determination of its molecular weight in previous rodent studies *in vivo* [17] and human clinical studies [18] (see Results and Discussion for details), we devised a 99% ^13^C-labeled PEG agent with an average molecular weight of 40,000 Da (^13^C-PEG40,000) and investigated its feasibility for tumor detection.

## Materials and Methods

### ^13^C-PEG

^13^C-enriched PEG using [1,2-^13^C]ethylene oxide (Cambridge Isotope Laboratories, MA) as a raw material was custom designed for use in this study. ^13^C-PEG40,000 and ^13^C-PEG7,000 were synthesized by Meisei Chemical Works, Ltd. (Kyoto, Japan) and DJK (Yokohama, Japan), respectively. The polydispersity value for the ^13^C-PEG40,000 was 1.47.

### *In vitro* NMR spectroscopy

NMR spectroscopy was conducted using a Bruker Avance III 400 spectrometer with a 9.4 T magnet. Measurements were performed with ^1^H irradiation to achieve ^1^H-^13^C nuclear Overhauser effect (NOE) and proton decoupling (WALTZ16). When a degree of the NOE effect was investigated, NMR measurements of ^12^C-based PEG40,000 were carried out with and without the ^1^H irradiation for NOE. Concentration was set at 50 mg/mL for the experiment. 5 mM of [1-^13^C]alanine was added as an internal standard.

For comparison of the ^13^C-NMR spectrum of PEG with those of other hydrophilic polymers, we measured intrinsic ^13^C signals of non-^13^C-enriched polymers (∼1% of carbon atoms in natural abundance) since no ^13^C-enriched polymer was available except for ^13^C-PEG40,000. PEG40,000 was purchased from NOF corporation (Tokyo, Japan); Dextran40,000, poly-L-lysine ∼50,000, PEG500,000, and Dextran200,000 from Wako (Osaka, Japan); and bovine serum IgG from Sigma. Concentrations of the smaller ^12^C-polymers (PEG40,000, Dextran40,000 and poly-L-lysine ∼50,000) were 5 mg/mL while the larger ^12^C-polymers (PEG500,000 and Dextran200,000) and IgG were 10 mg/mL, with phosphate-buffered saline (PBS), pH 7.4, including 20% D_2_O. All measurements were performed using a 5-mm test tube at 24°C and with a 1.36 s acquisition time, a 2.0 s relaxation delay, and a 30° flip angle (8.5 µs for 90° pulse width), and consisted of 3200 scans. 1 mM of [1-^13^C]alanine was used as an internal standard. Calculation of signal-to-noise ratio was conducted using Bruker TOPSPIN 2.1 software, with estimation of signal half-width manually performed using the software with an expanded plot.

Evaluation of T_1_ (for Table S1) was performed using the inversion recovery method and Bruker TOPSPIN 2.1 software was used for subsequent data analysis. The concentration was set at 20 mg/mL for ^13^C-PEG40,000 and 50 mg/mL for ^12^C-based polymers.

### Fluorescently labeled PEG40,000

Amino-terminal PEG40,000 (SUNBRIGHT MEPA-40T, NOF) and tetramethyl rhodamine (TMR) succinimidyl ester mixed isomer (Invitrogen) were used for labeling of PEG40,000 with tetramethyl rhodamine (TMR-PEG40,000). 50 mg of amino-terminal PEG40,000 and 5 mg of TMR succinimidyl ester were mixed for 20 h at room temperature in 0.5 M bicarbonate buffer, pH 8.3, and purified by Superdex75 gel filtration column. The collected TMR-PEG40,000 fraction was concentrated using Amicon Ultra centrifuge filters (cut-off Mw 10,000 Da) before use. The labeling efficiency was ∼100% as estimated by comparison of concentration of TMR with that of PEG, which were determined by intensities of absorbance and ^13^C-NMR signal, respectively.

### Manipulation of cells

C26 (murine colon adenocarcinoma 26 cell line) and Miapaca2 (human pancreas cancer cell line) were obtained from a cell line collection of Otsuka Pharmaceutical Co., Ltd. Caki2 (human Caucasian kidney carcinoma cell line) was purchased from the European Collection of Cell Cultures. T24 (human bladder cancer cell line) was supplied by the Cell Resource Center for Biomedical Research, Institute of Development, Aging and Cancer at Tohoku University. Miapaca2 and T24 were genetically authenticated using STR analysis (Promega, October 2012). C26 cells were maintained in RPMI1640, T24 and Caki2 cells in McCoy's 5A, and Miapaca2 cells in D-MEM/Ham's F-12 with 10% fetal bovine serum (FBS), 50 U/mL of penicillin, and 50 µg/mL of streptomycin (a penicillin/streptomycin mixture) in a humidified atmosphere containing 5% CO_2_ at 37°C.

### Ethics statement and preparation of tumor-bearing mice

Male BALB/c and BALB/c nude mice were purchased from Japan SLC, Inc. All procedures were performed in accordance with the guidelines of Science Council of Japan (Guidelines for Proper Conduct of Animal Experiments) and the guidelines of the Animal Care and Use Committee of Otsuka Pharmaceutical Co., Ltd, and approved by the company committee (OA0057). All surgery was performed under isoflurane anesthesia to relieve pain. All mice were housed in a specific pathogen free facility under the standard conditions recommended in the guidelines and used for experiments before the tumor size became unnecessarily large to minimize suffering. We subcutaneously inoculated 1×10^6^∼10^7^ C26 cells or human cancer cells (Miapaca2, Caki2, T24) suspended in Hank's Balanced Salt Solution (HBSS) into the backs of BALB/c mice or nude mice, respectively. The resulting animal models were not subjected to experimentation until the tumor volume had exceeded ∼100 mm^3^. The total number of mice used for this study was 59.

### Preparation and fluorescence microscopy of frozen C26 tumor sections

TMR-PEG40,000 was intravenously injected into C26 tumor-bearing mice at a dose of 250 mg/kg. The mice were sacrificed 120 h post-injection and tumors were extirpated for use in the preparation of frozen sections. Extirpated tumors were embedded in an OCT compound followed by immediate freezing using liquid nitrogen. Frozen sections were cut at a 5-µm thickness using a microtome, fixed with acetone on slide glasses, stained using 1 µg/mL of Hoechst 33342 (Dojin, Japan), and embedded using Fluoromount (Diagnostic BioSystems, USA) with coverslips. The reproducibility was checked by conducting three independent experiments.

Fluorescence microscopy of the frozen sections was performed using a KEYENCE BZ-9000 system (Osaka, Japan) with a 100× Nikon Plan Apo oil lens with a 1.4 numerical aperture.

### Evaluation of concentration of ^13^C-PEG in blood circulation

^13^C-PEG was intravenously injected into BALB/c mice at a dose of 92 mg/kg. After 5 µl of blood had been collected from the tail vein at each sampling time, the samples were immediately added to 495 µl of PBS containing 10 mM of EDTA and then centrifuged at 18,000× *g* for 10 min to remove debris. The concentration of ^13^C-PEG was estimated using ^13^C-NMR and comparing the signal intensity of ^13^C-PEG with that of 1 mM [1-^13^C]alanine as an internal standard (signal intensity of 1 mg/mL ^13^C-PEG was 125.6 when that of 1 mM [1-^13^C]alanine was set at 1.0). 3 mice were used to obtain the blood circulation data for each ^13^C-PEG (^13^C-PEG40,000 and ^13^C-PEG7,000).

### Evaluation of ^13^C-PEG40,000 accumulation in tumor and other tissues

^13^C-PEG40,000 was intravenously injected into the tail vein of C26-tumor bearing mice at a dose of 92 mg/kg. After mice were sacrificed and dissected at various periods after intravenous injection, liver, kidney, spleen, pancreas, and tumor tissue were homogenized in phosphate-buffered saline (PBS). Supernatants were collected after centrifugation for ^13^C-PEG40,000 quantification using NMR spectroscopy. Concentration of ^13^C-PEG40,000 in the supernatant was estimated from analysis of the ^13^C-NMR spectra by comparison of the signal intensity of ^13^C-PEG with that of 1 mM [1-^13^C]alanine.

### *In vivo* MR measurements

*In vivo* MR data of mice were acquired with a 7 T Unity Inova System (Agilent, Santa Clara, USA). A custom-made ^1^H- and ^13^C-dual-tuned (300 MHz and 75 MHz, respectively) volume coil 40 mm in diameter and 100 mm in length was used for ^1^H MRI and ^13^C chemical shift imaging (CSI). Mice were anesthetized throughout MR measurement under spontaneous breathing with 1.5% isoflurane carried in 50% O_2_ through a face mask. Under anesthesia, mice were fixed on a cradle in a supine position for insertion of the dual-tuned RF coil. Multislice ^1^H MR images in the coronal plane were acquired using a gradient echo sequence at a 300-ms repetition time (TR), a 3-ms echo time (TE), a 100×50 mm^2^ field of view (FOV), a 2-mm slice thickness, and a 256×128 matrix. An image including the implanted tumor and major organs was selected as a reference image.

^13^C-CSI data were obtained using the same field of view (FOV) used for ^1^H imaging (100×50 mm^2^) in the coronal plane without slice selection. Free induction decay (FID) data were acquired under ^1^H irradiation to achieve ^1^H-^13^C NOE and ^1^H decoupling, with a 250-ms repetition time (TR) and 64 acquisitions for 16×8 phase encoding steps. Data processing by 3D Fourier transformation with zero-filling and magnitude calculation allowed for achievement of 64×32 power spectra and subsequent construction of 2-dimensional PEG images with 64×32 matrices by integration of the peak areas. Each ^13^C image was independently created using an 8-bit scale under the assumption that a pixel of the lowest intensity in a ^13^C image had an intensity of 0 and a pixel of the highest intensity an intensity of 255. The total acquisition time of one data set was 34 min. The reproducibility was checked by several independent experiments for each tumor model (n = 6 for C26 tumor model, n = 4 for Miapaca2 model, n = 3 for Caki2 and T24 models, respectively).

## Results and Discussion

### Molecular weight setting used for ^13^C-PEG synthesis

The molecular weight setting used for ^13^C-PEG synthesis was based on 3 previous findings. First, the accumulation of macromolecules in tumor requires a prolonged period of circulation [15], [16]. Second, as demonstrated by Yamaoka *et al.* [17], the half-life of PEG in circulation strongly depends on its molecular size, as PEG molecules that are smaller than an established cut-off value of a glomerular pore size are rapidly excreted by renal clearance. Specifically, Yamaoka *et al.* [17] found that the half-life values of PEG molecules with a molecular weight greater than ∼50,000 Da do not change and reach a plateau in the profile of molecular weight vs. half-life in circulation. Third, PEG molecules with a molecular weight of 40,000 Da have been intravenously administered effectively to humans in a camptothecin-conjugated form [18]. Therefore, the optimal weight-averaged molecular weight of ^13^C-PEG for tumor detection was set at 40,000 Da. ^13^C-PEG synthesized to have a weight-averaged molecular weight of 7,000 Da (^13^C-PEG7,000) was used as a control.

### ^13^C spectral features of polyethylene glycol

**Figure 1:**
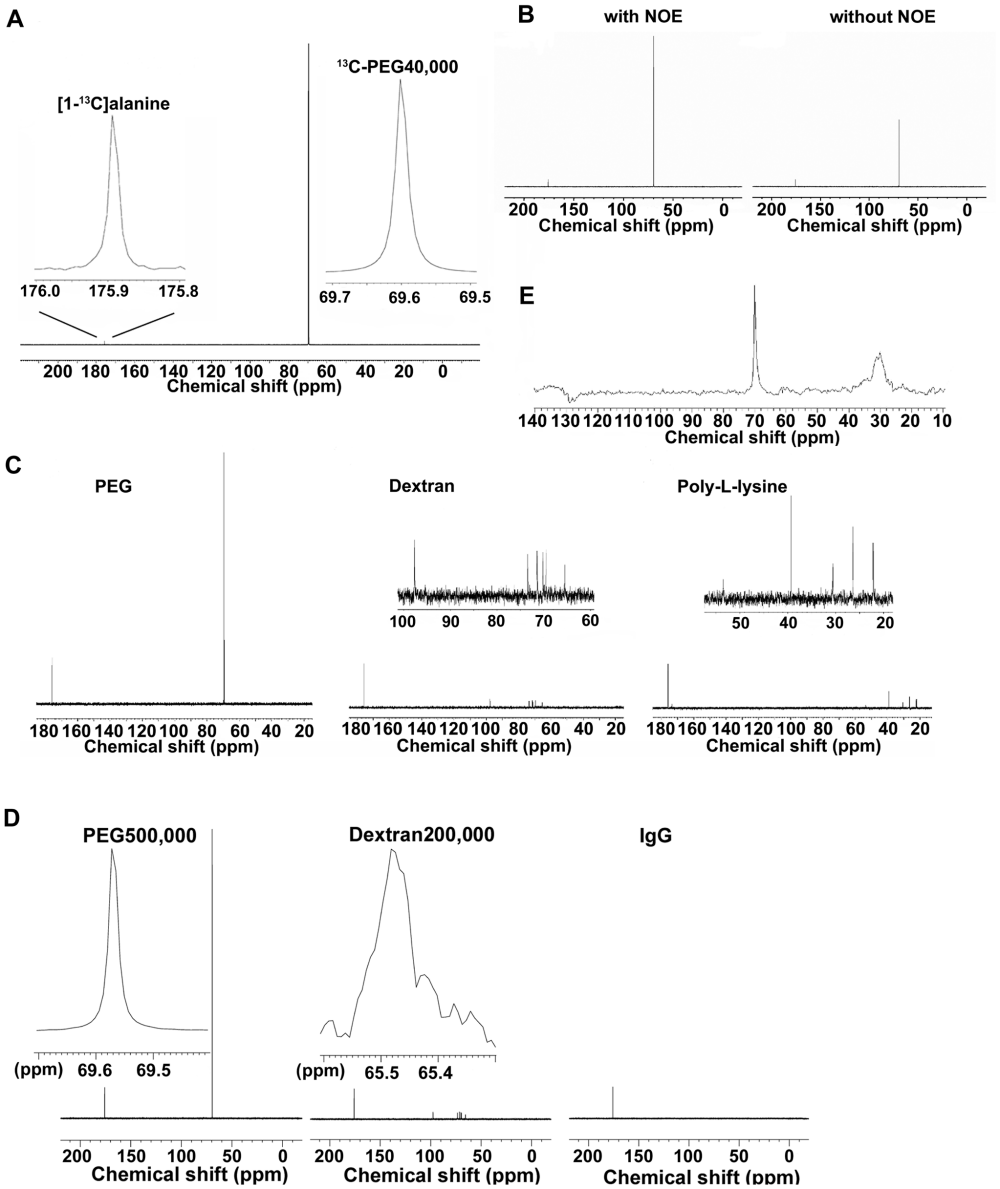
^13^C spectra of PEG and other hydrophilic polymers. (A) ^13^C spectrum of 1 mg/mL of ^13^C-PEG40,000 (25 µM). 1 mM [1-^13^C]alanine was used as an internal standard. *Inset*, ^13^C signals of [1-^13^C]alanine and ^13^C-PEG40,000 revealed by magnification. (B) *Left panel*, ^13^C spectrum of ^12^C-based PEG40,000 measured with proton irradiation to induce NOE from protons to carbon atoms. *Right panel*, ^13^C spectrum of ^12^C-based PEG40,000 measured without proton irradiation for NOE. Internal standard signal of [1-^13^C]alanine was observed at 175.9 ppm. (C) ^13^C spectra of ^12^C-based polymers. The signal of [1-^13^C]alanine used as an internal standard was detected at 175.9 ppm in each spectrum. *Left panel*, a signal of PEG40,000 was detected at 69.6 ppm. *Middle panel*, 6 signals from each carbon atom in dextran with an average molecular weight of 40,000 Da were detected at 65.5, 69.5, 70.1, 71.4, 73.4, and 97.7 ppm. *Inset*, magnification of the spectrum region from 60 to 100 ppm. *Right panel*, 6 signals from each carbon atom in poly-l-lysine with an average molecular weight of ∼50,000 Da were detected at 22.1, 26.4, 30.6, 39.3, 53.4, and 173.7 ppm. *Inset*, magnification of the spectrum region from 25 to 55 ppm. (D) ^13^C spectra of ^12^C-based polymers with a large molecular weight and bovine serum IgG. The signal of [1-^13^C]alanine used as an internal standard was detected at 175.9 ppm in each spectrum. *Left panel*; the signal of PEG with an average molecular weight of 500,000 Da (69,6 ppm). *Inset*, magnification of the PEG signal. *Middle panel*; 6 signals from each carbon atom in dextran with an average molecular weight of 200,000 Da. *Inset*, magnification of the signal at 65.5 ppm. *Right panel*; ^13^C signaling from IgG was not observed. (E) ^13^C spectrum of the entire body of a mouse 1 h after intravenous injection of 250 mg/kg of ^13^C-PEG40,000.

^13^C-PEG40,000 exhibited a very characteristic ^13^C-NMR signal, specifically a single, strong, and sharp signal at 69.6 ppm (Fig. 1A). Despite the much larger mass of ^13^C-PEG40,000, the ^13^C-PEG40,000 signal had a half-width analogous to that of [1-^13^C]alanine at 175.9 ppm as an internal standard (2.3±0.19 Hz and 1.7±0.17 Hz for ^13^C-PEG40,000 and [1-^13^C]alanine, respectively. n = 10). The T_1_ of the ^13^C-PEG40,000 carbon atoms was 591.8±15.4 ms (n = 4), which was sufficiently rapid to increase the number of repetitions within a restricted measurement period.

The strong signal of PEG largely depended on the Nuclear Overhauser Effect (NOE) from protons to carbon atoms as shown in Fig. 1B. The signal area intensity of PEG was approximately 3 times larger with the NOE irradiation. This is because all of the carbon atoms in PEG have an identical chemical structure in a manner that a carbon atom forms chemical bonds with 2 protons, 1 carbon atom and 1 oxygen atom. The unique chemical structure led to the efficient NOE from the adjacent 2 protons to each ^13^C nucleus. We compared a ^13^C NMR spectrum of ^12^C-based PEG with ^13^C NMR spectra of other ^12^C-based representative hydrophilic polymers with comparable molecular weights (40,000∼50,000 Da), dextran and poly-L-lysine, to investigate the advantages of ^13^C signal of PEG (Fig. 1C and summarized in Table S1). The most striking feature in the ^13^C NMR spectrum of PEG was the single signal, while the signals of ^13^C nuclei of dextran and poly-L-lysine were dispersed at 6 different chemical shifts. The single signal arises from the unique chemical structure consisting of -CH_2_CH_2_O- repetitions in which all ^13^C nuclei have an identical chemical environment. The non-dispersed signal of PEG largely contributed to the strong signal of PEG. Furthermore, the signal half-width of the ^13^C nuclei of PEG was smaller than those of other hydrophilic polymers. The signal half-width was ∼2.2 Hz for PEG, while those of other polymers ranged from 2.8 to 8.5 Hz (Table S1). The samller half-width also contributed to the strong signal of PEG. Because a signal half-width in NMR spectra is generally inversely proportional to a rate of molecular motion, the small half-width of PEG is consistent with the notion that PEG exhibits enhanced structural flexibility and hydrophilicity [19]. We measured T_1_ values of a certain ^13^C nuclei in the hydrophilic polymers. The T_1_ value of the ^13^C nuclei of PEG was ∼560 ms, while those of the dextran signal at 97.7 ppm and the poly-L-lysine signal at 39.9 ppm, which were the highest signal among the 6 signals of the molecules, exhibited smaller (∼270 ms) and comparable (∼580 ms) values, respectively. Although we could not disclose the extent of the contribution of the T_1_ to the strong signal of PEG, the moderately short T_1_ value that are applicable to all ^13^C nuclei in the molecule might partly contribute to the signal strength because the short T_1_ would contribute to increase in the cumulative number of NMR signal acquisition within a restricted measurement period. Taken together, the strong signal of PEG can be attributed to the feature that all the ^13^C nuclei in PEG share the properties of signal sharpness, non-signal dispersion, moderately short T_1_ and the efficient NOE from adjacent protons.

Interestingly, signal sharpness was maintained among PEG molecules of much larger mass, specifically those with an average molecular weight of 500,000 Da (signal half-width, 2.0±0.07 Hz, n = 4), indicating that the flexibility of each carbon atom in a PEG molecule is maintained, regardless of its mass in aqueous solution (Fig. 1D). In contrast, large dextran molecules with an average molecular weight of 200,000 Da generally exhibited broad signals, while no ^13^C signaling could be detected from bovine serum IgG (molecular weight of ∼150,000 Da, Fig. 1D). These results, in addition to the detection of sharp and strong ^13^C-PEG40,000 signals (∼70 ppm) readily distinguishable from the large and broad signals of endogenous fat (∼30 ppm) *in vivo* (Fig. 1E), support the superiority of PEG for the detection of ^13^C signals.

### Accumulation and retention of ^13^C-PEG40,000 in solid tumors in mice

**Figure 2:**
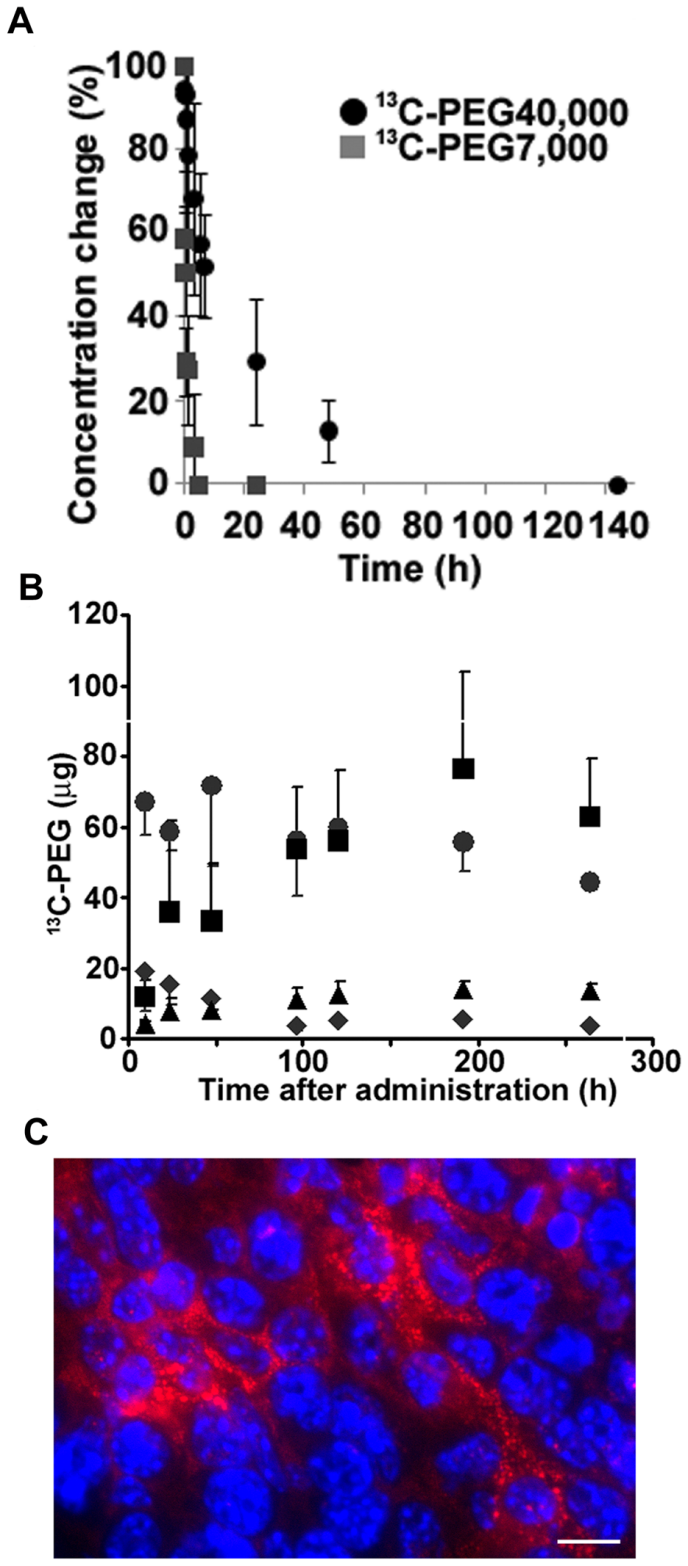
Concentration profiles of ^13^C-PEGs in circulation and the accumulation of ^13^C-PEG40,000 in each tissue. (A) Concentration change in ^13^C-PEGs of different molecular weights (black circles, ^13^C-PEG40,000; gray squares, ^13^C-PEG7,000) in blood circulation of mice after i.v. injection of 92 mg/kg. The concentration at the first sampling point after injection (2 min) was set at 100%. Data presented are the mean and standard deviation values calculated from the results of 3 independent experiments. (B) A graph showing the time dependence of the absolute amount of ^13^C-PEG40,000 accumulated in C26 tumor and normal tissues 9, 24, 48, 96, 120, 192, and 264 h after intravenous injection of 92 mg/kg in 3, 6, 6, 5, 6, 4, and 4, mice, respectively. *Squares*; tumor tissue, *circles*; liver tissue, *triangles*; spleen tissue, *diamonds*; kidney tissue. Data presented are the mean and standard deviation. (C) A representative high-power micrograph of an area from a frozen section of C26 tumor 120 h post-injection of TMR-PEG40,000, being obtained by merging a micrograph of Hoechst 33342 fluorescence (dye for nuclear stain, shown in blue) and TMR-PEG40,000 fluorescence (shown in red). Scale bar = 10 µm.

To further examine the utility of ^13^C-PEG40,000 for tumor detection *in vivo*, the concentration profile of ^13^C-PEG40,000 in circulation after intravenous (i.v.) injection into normal BALB/c mice was first examined. In contrast to the concentration of smaller ^13^C-PEG molecules with an average molecular weight of 7,000 Da (^13^C-PEG7,000), which exhibited an abrupt decrease, the concentration of ^13^C-PEG40,000 in normal BALB/c mice was retained for an extended period in circulation (Fig. 2A). The half-lives of the ^13^C-PEG in circulation were calculated to be about 510 min and 11 min for ^13^C-PEG40,000 and ^13^C-PEG7,000, respectively. This difference in retention period in circulation can be explained by the difference in renal clearance efficiency among distinctly sized PEGs, as previously reported [17], [20]. As expected from the extended circulation of ^13^C-PEG40,000, tumor tissue was found to have a higher accumulation efficiency (∼6% injected dose/g tumor) compared to normal tissues (∼2% injected dose/g tissue for liver, kidney, spleen and pancreas, Figure S1) at a relatively early period post-i.v. injection (9 h–48 h) in C26-tumor-bearing mice. This result can be attributed to the enhanced leakiness of tumor capillaries, which is widely recognized [15], [16], [20].

The % injected dose/g value largely depends on tumor weight because the denominator is the tumor weight. However, as the tumor weight continued to increase post-injection in this tumor model, the % injected dose/g value could have led to a misunderstanding of the tendency for ^13^C-PEG40,000 to accumulate in tumor tissue. Thus, time-dependent accumulation of ^13^C-PEG40,000 in tumor tissue was not evaluated by % injected dose/g but rather by the absolute amount of ^13^C-PEG40,000 accumulated in the tumor. As shown in Fig. 2B, the absolute amount of ^13^C-PEG40,000 in the tumor gradually increased with time after i.v. injection until reaching a maximum at 192 h post-injection, where its level remained until 264 h post-injection. This result demonstrates the tendency of ^13^C-PEG40,000 to be retained in tumor tissue over an extended period. The amount of ^13^C-PEG40,000 accumulated in the liver, the largest organ in mice and responsible for accumulation and metabolism of unnecessary substances, was comparable to that accumulated in tumor tissue around 100 h post-injection. However, the time-dependence profile of ^13^C-PEG40,000 accumulated in liver tissue was obviously distinct from that of tumor tissue, gradually decreasing with time after i.v. injection and being exceeded by the amount accumulated in tumor tissue after 120 h post-injection (Fig. 2B). On the other hand, the amount of ^13^C-PEG40,000 accumulated in spleen and kidney tissue was much less than that accumulated in tumor tissue throughout the experimental period.

The long-term retention in tumor indicates that PEG molecules are associated with tumor cells after extravasation into tumor interstitium. Indeed, localization of endocytosed fluorescently labeled PEG40,000 (TMR-PEG40,000) to lysosomes was observed as a dot-like manner in the *in vitro* experiments using C26 cells (Figure S2). In order to evaluate the localization of PEG molecules in the tumor *in vivo*, microscopic experiments using TMR-PEG40,000 with C26-tumor bearing mice were conducted. In a frozen section of a C26 tumor that had been isolated at 120 h post-i.v. injection of TMR-PEG40,000, fluorescence of TMR-PEG40,000 was observed from the region just adjacent to the nuclei and throughout the cytoplasm in tumor cells in a dot-like manner (Fig. 2C), suggesting that TMR-PEG40,000 molecules extravasating into tumor interstitium had been incorporated into tumor cells *in vivo*, as observed in the preceding *in vitro* experiments (Figure S2).

### Visualization of solid tumors in mice using ^13^C-PEG40,000

**Figure 3:**
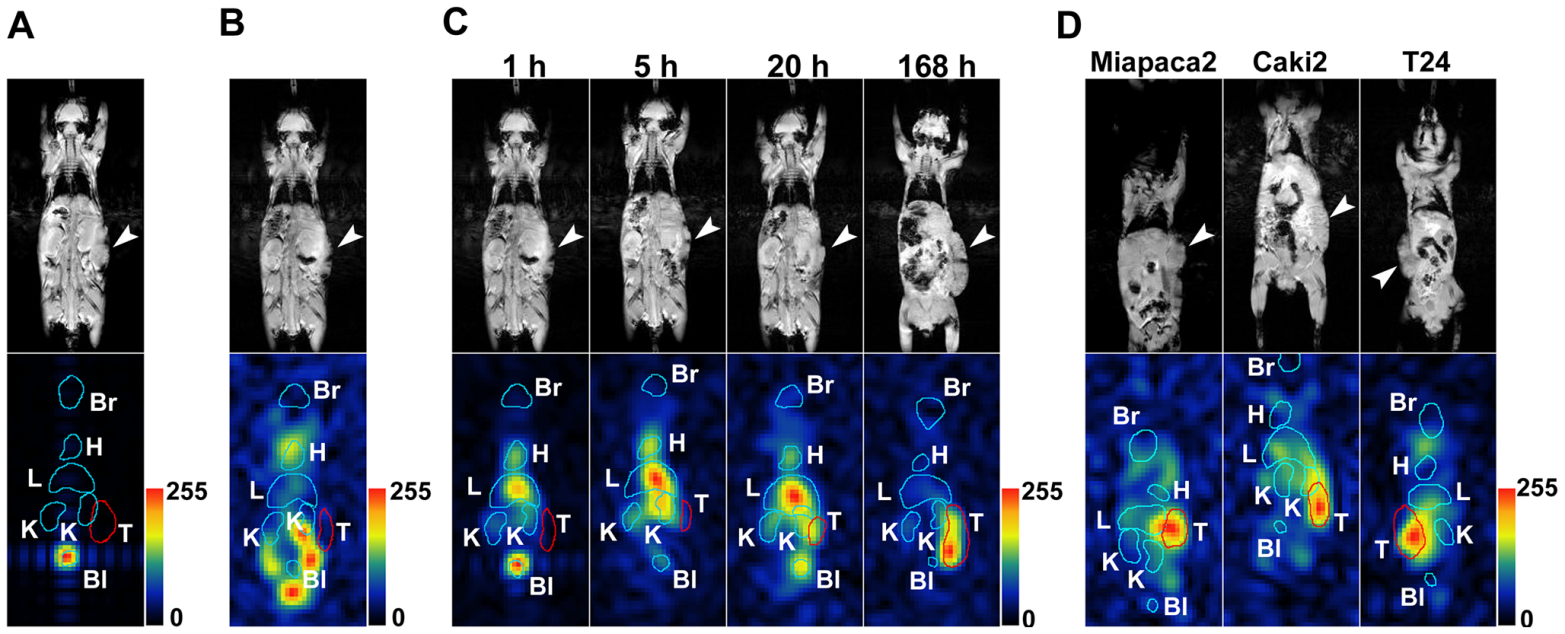
Representative ^13^C-PEG40,000 images of tumor-bearing mice. The upper and lower panels show a gradient-echo image and the corresponding ^13^C image, respectively. Each image represents the FOV of 100 mm in length and 50 mm in width. No additional contrast enhancement was performed for any ^13^C images, and thus all faithfully represent raw data with respect to intensity. Intensity scale bars represent linear change in intensity from 0 (black) to 255 (red). An arrowhead in each gradient-echo image shows the tumor position. Blue and red lines in ^13^C images represent the rough position of each tissue, with the positions of the tumors highlighted by a red line. Br, H, L, K, T, and Bl in each ^13^C image indicate the brain, heart, liver, kidney, tumor, and bladder, respectively. (A) Biodistribution image of ^13^C-PEG7,000 in a C26 tumor-bearing mouse 1 h after intravenous injection. (B) ^13^C image of endogenous fat created from the same data set used in the 1 h data in (C). The broad and intense endogenous fat signal shown in Fig. 1E was used to create the ^13^C image. (C) Sequential ^13^C images showing the time dependence of the biodistribution of ^13^C-PEG40,000 in a C26 tumor-bearing mouse. (D) Biodistribution images of ^13^C-PEG40,000 in each xenograft of human cancer cell line 96 h to 120 h after intravenous injection. Miapaca2, Caki2, and T24 are well-known human cancer cell lines established from cancer cells in the human pancreas, kidney, and bladder, respectively.

To validate the potential of ^13^C-PEG40,000 in ^13^C spectroscopic imaging for detecting tumors subcutaneously transplanted into mice, ^13^C chemical shift imaging (CSI) was performed to obtain biodistribution images of ^13^C-PEG with a custom-made coil. Representative images from each experiment are shown in Fig. 3. In the investigation of the biodistribution of ^13^C-PEG7,000 (Fig. 3A), ^13^C signals at 1 h post-injection were only detected from the bladder, and no clear biodistribution pattern except for that in the bladder was observed after 1 h. Such limited distribution to the bladder appeared to reflect rapid renal excretion, as can be inferred from the rapid decline of ^13^C-PEG7,000 in circulation (Fig. 2A). On the other hand, ^13^C-PEG40,000 displayed a broad biodistribution pattern that was clearly distinct from that of endogenous fat (Fig. 3B) and that changed dramatically in a time-dependent manner (Fig. 3C). At 1 h post-injection, intense ^13^C signals were detected from the liver and bladder, while modest and weak ^13^C signals were detected from the heart and kidney, respectively. This distribution pattern may be explained by the hypothesis that a population of smaller-sized ^13^C-PEG40,000 molecules below the glomerular threshold was being excreted by renal clearance while a larger-sized population above the threshold was being retained in circulation. At 5 h post-injection, strong ^13^C signals were detected from the liver but no signals from the tumor. At 20 h post-injection, the ^13^C signaling from the liver remained intense as modest signaling from the tumor began to appear. At 168 h post-injection, the signals from the tumor had become by far the strongest in the entire body. It should be noted that the degree of signal intensity in one of the 4 images in Fig. 3C cannot be directly compared with those in the other 3 images since these sequential ^13^C images were obtained through independent procedures of MR measurement including specimen handling at each time period. It should also be noted here that signal intensity in a ^13^C image was defined not only by the ^13^C-PEG amount but was also markedly influenced by many other factors, such as magnetic field inhomogeneity, relative size ratio between the sizes of a voxel and a region of interest, and motion of individual tissues. Therefore, the time-dependent accumulation trends of ^13^C-PEG40,000 in each tissue shown in Fig. 2B do not necessarily correspond to the time-dependent change in ^13^C images shown in Fig. 3C. Especially in the last image at 168 h post-injection in Fig. 3C, the ^13^C signal from the liver tissue may have weakened, possibly because of several factors described above.

Subsequent *in vivo* examination of the effectiveness of ^13^C-PEG40,000 in the detection of human tumors revealed that ^13^C-PEG40,000 enabled visualization of representative human tumors subcutaneously implanted into nude mice with a comparable tumor-to-normal tissue ratio as that observed in the C26 model (Fig. 3D). Although background signals in thigh muscles are various depending on individual mice, the tumor-to-muscle ratios were at least more than ∼5 for a number of mice we tested and the tumor location could be readily identified in an entire mouse body. These data clearly demonstrate the possibility of utilizing ^13^C-PEG in practical human diagnosis.

Success in visualizing tumors using ^13^C-PEG largely depends on the sharp and strong ^13^C signal of PEG. As described in the section of ^13^C spectral features of PEG, it is generally recognized that PEG exhibits enhanced structural flexibility and hydrophilicity [19], and so it is likely that the sharpness of the ^13^C signal is because of these characteristics of PEG in an aqueous solution. The flexibility of PEG will contribute to canceling out broadening of the signal as the molecular weight increases. Therefore, broadening and attenuation of the signal would not occur. In fact, we demonstrated that the signal half-width of PEG with much larger molecular weight (∼500,000 Da) did not broaden out as compared with that with molecular weight of 40,000 Da. The hydrophilicity of PEG will also be important for the sharpness of the signal, especially *in vivo*. The formation of a layer of water molecules surrounding PEG resulting from its high hydrophilicity was previously reported [21]. The protection of the water layer would prevent PEG from interacting with other molecules in an *in vivo* environment. Therefore, PEG will maintain free motion even in a highly viscous environment *in vivo*, which will minimize the broadening of the ^13^C signal.

^12^C-based PEGs have been widely tested in the modification of therapeutic and diagnostic agents to improve their stabilities in the blood circulation [22], [23]. As being currently performed using ^12^C-based PEGs, ^13^C-PEG can be also used as a modification polymer for therapeutic nanoparticles such as micelles or lyposomes, and also for diseased-site-specific therapeutic antibodies to improve their stabilities in the blood circulation. If the ^13^C-PEG would be utilized in such a manner, a multifunctional agent that enables medical treatments and site-specific imaging diagnoses at the same time could be implemented. Therefore the ^13^C-PEG strategy could be useful in a broad area of medicine.

The most characteristic feature of the ^13^C-PEG strategy is the lack of temporal limitations. Because of this property, we are able to wait not only until ^13^C-PEG sufficiently accumulated in tumors, but also until its concentration in circulation adequately declined after administration of the tracer. This is the most beneficial feature of using a stable isotope. This property would enable diagnoses to be made in renal and urinary systems, in which hyperpolarized and/or radiolabeled tracers may work insufficiently because of the substantial background of the tracer itself. Therefore, the ^13^C-PEG strategy could be utilized to supplement methods that use hyperpolarized and/or radiolabeled tracer strategies, which could introduce a novel application of MRI into the field of tumor detection.

## Conclusions

In this study, we have succeeded in clearly visualizing the tumors of tumor-bearing mice by using ^13^C-enriched PEG in ^13^C spectroscopic imaging. The ^13^C-PEG strategy relies on the strong and sharp ^13^C nuclear magnetic resonance signal of ^13^C-PEG. This strategy has provided a novel usage of PEG as a tracer.

## Supporting Information

Figure S1 **Accumulation efficiency of ^13^C-PEG40,000 in tumor and normal tissues of C26-tumor bearing mice.** Bars represent accumulation efficiency of ^13^C-PEG40,000 in the designated tissues at 9, 24, and 48 h after i.v. injection of 92 mg/kg. L, K, S, T, and P represent liver, kidney, spleen, tumor, and pancreas, respectively. Data presented are the mean and standard deviation values calculated from the results of 3 (9 h) or 6 (24, 48 h) independent experiments.
(TIF)

Figure S2 **Cellular uptake of TMR-PEG40,000** ***in vitro*** **observed by fluorescence microscopy.** Co-localization with TMR-PEG40,000 and lysotracker (a fluorescent dye for lysosomal stain, Life technologies) in C26 cells was observed. The fluorescence microscopic observation was conducted after the incubation of C26 cells with TMR-PEG40,000 at a concentration of 10 µM for 5 h at 37°C. Fluorescence of lysotracker is shown in green and that of TMR-PEG40,000 is shown in red. Scale bar = 10 µm.
(TIF)

Table S1 **Spectral properties of ^13^C signals of PEG40,000 and other hydrophilic polymers with similar molecular weight.**
(DOC)

Checklist S1 **The ARRIVE Guidelines Checklist.**
(DOC)
