# Supplementary figures and images for: A Polymer-Based Magnetic Resonance Tracer for Visualization of Solid Tumors by 13C Spectroscopic Imaging
Source: PLoS One. 2014 Jul 9;9(7):e102132. doi: 10.1371/journal.pone.0102132 (PMC4090184; doi:10.1371/journal.pone.0102132)

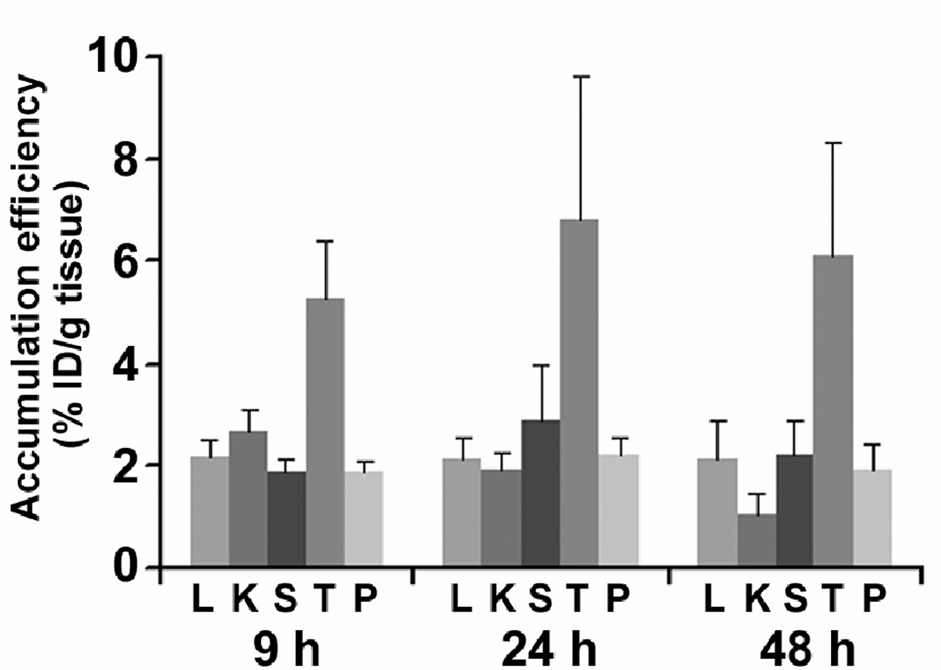

Supplement: Figure S1 — Accumulation efficiency of 13C-PEG40,000 in tumor and normal tissues of C26-tumor bearing mice. Bars represent accumulation efficiency of 13C-PEG40,000 in the designated tissues at 9, 24, and 48 h after i.v. injection of 92 mg/kg. L, K, S, T, and P represent liver, kidney, spleen, tumor, and pancreas, respectively. Data presented are the mean and standard deviation values calculated from the results of 3 (9 h) or 6 (24, 48 h) independent experiments. (TIF) [file pone.0102132.s001.tif]

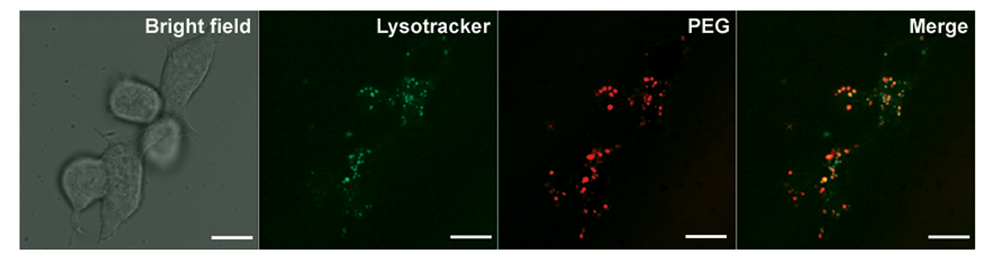

Supplement: Figure S2 — Cellular uptake of TMR-PEG40,000 in vitro observed by fluorescence microscopy. Co-localization with TMR-PEG40,000 and lysotracker (a fluorescent dye for lysosomal stain, Life technologies) in C26 cells was observed. The fluorescence microscopic observation was conducted after the incubation of C26 cells with TMR-PEG40,000 at a concentration of 10 µM for 5 h at 37°C. Fluorescence of lysotracker is shown in green and that of TMR-PEG40,000 is shown in red. Scale bar = 10 µm. (TIF) [file pone.0102132.s002.tif]
