# Supplementary material for: A Polymer-Based Magnetic Resonance Tracer for Visualization of Solid Tumors by 13C Spectroscopic Imaging
Source: PLoS One. 2014 Jul 9;9(7):e102132. doi: 10.1371/journal.pone.0102132 (PMC4090184; doi:10.1371/journal.pone.0102132)
Supplement: Table S1 — Spectral properties of 13C signals of PEG40,000 and other hydrophilic polymers with similar molecular weight. (DOC) [file pone.0102132.s003.doc]

Table S1.

Spectral properties of ^13^C signals of PEG40,000 and other hydrophilic polymers with similar molecular weight.

PEG (Mw: 40,000 Da)

Dextran (Mw: 40,000 Da)

Poly-l-lysine (Mw: ~50,000 Da)

Number of signals

1

6

6

Concentration of

carbon atoms (M) ^b^

0.23

0.19

0.23

Signal area

intensity (a.u.) ^a, c^

7.5 ± 0.34

Max. 0.47 ± 0.12 (97.7 ppm)

Max. 0.63 ± 0.01 (39.3 ppm)

Total 2.6 ± 0.40

Total 2.7 ± 0.11

Signal half-width (Hz) ^a, d^

2.2 ± 0.07

Min. 5.1 ± 0.12 (70.1 ppm)

Min. 2.8 ± 0.07 (39.3 ppm)

Max. 6.9 ± 0.56 (65.5 ppm)

Signal-to-noise ratio ^a, e^

2.6 × 10^2^ ± 3.0

7.8 ± 1.0 (97.7 ppm)

1.7 × 10 ± 0.1 (39.3 ppm)

Max. 8.5 ± 1.7 (53.4 ppm)

T_1_ (ms) ^a^

563.4 ± 2.5

268.9 ± 8.8 (97.7 ppm)

575.4 ± 7.1 (39.3 ppm)

^d^ For dextran and poly-l-lysine, the values of the minimum and the maximum of the 6 signals are denoted.

^b^ These values were calculated from the molecular weight of the repetition unit in each polymer and the number of carbon atoms in the unit. The repetition units were CH_2_CH_2_O, C_6_H_10_O_5_, and C_6_H_12_N_2_O for PEG, dextran, and poly-l-lysine, respectively. The molecular weights of the repetition unit were 44, 162, and 128 for PEG, dextran, and poly-l-lysine, respectively. The concentration of each polymer was 5 mg/mL.

^c^ Signal area intensity was calculated with the assumption that the intensity of [1-^13^C]alanine was 1.0.

For dextran and poly-l-lysine, the values of total intensity and maximum intensity of the 6 signals are denoted.

^a^ The values of signal area intensity, signal half-width, and signal-to-noise ratio are shown as the mean ± standard deviation values calculated from the results of 3 independent experiments. The values of T_1_ are shown as the mean ± standard deviation values calculated from the results of 2 independent experiments. The values in parentheses indicate the values of the signals used for the calculations.

^e^ For dextran and poly-l-lysine, the highest value of the 6 signals is indicated.
